# Abscopal effect of radiotherapy combined with immune checkpoint inhibitors

**DOI:** 10.1186/s13045-018-0647-8

**Published:** 2018-08-16

**Authors:** Yang Liu, Yinping Dong, Li Kong, Fang Shi, Hui Zhu, Jinming Yu

**Affiliations:** 1 School of Medicine and Life Sciences, University of Jinan-Shandong Academy of Medical Sciences, Jinan, Shandong China; 2 Department of Radiation Oncology, Shandong Cancer Hospital affiliated to Shandong University, Shandong Academy of Medical Sciences, 440 Jiyan Road, Jinan, 250117 Shandong China

**Keywords:** Cancer, Radiotherapy, Immunotherapy, Abscopal effect

## Abstract

Radiotherapy (RT) is used routinely as a standard treatment for more than 50% of patients with malignant tumors. The abscopal effect induced by local RT, which is considered as a systemic anti-tumor immune response, reflects the regression of non-irradiated metastatic lesions at a distance from the primary site of irradiation. Since the application of immunotherapy, especially with immune checkpoint inhibitors, can enhance the systemic anti-tumor response of RT, the combination of RT and immunotherapy has drawn extensive attention by oncologists and cancer researchers. Nevertheless, the exact underlying mechanism of the abscopal effect remains unclear. In general, we speculate that the immune mechanism of RT is responsible for, or at least associated with, this effect. In this review, we discuss the anti-tumor effect of RT and immune checkpoint blockade and discuss some published studies on the abscopal effect for this type of combination therapy. In addition, we also evaluate the most appropriate time window for the combination of RT and immune checkpoint blockade, as well as the optimal dose and fractionation of RT in the context of the combined treatment. Finally, the most significant purpose of this review is to identify the potential predictors of the abscopal effect to help identify the most appropriate patients who would most likely benefit from the combination treatment modality.

## Background

Radiotherapy (RT) is a treatment for malignant tumors that has been used for the past century and has been applied to approximately 50% of all cancer patients [1–3], including patients with newly diagnosed cancers and those with persistent or recurrent tumors. Historically, radiation-induced deoxyribonucleic acid (DNA) damage, which leads to direct tumor cell death by the process of tumor cell apoptosis, senescence, and autophagy [4–6], is considered to be the major mechanism by which most solid tumors respond to clinical ionizing radiation [7]. Since these cytotoxic effects can also affect leukocytes, RT has been considered to be immunosuppressive. For example, the phenomenon of lymphopenia following RT has been observed in patients with solid tumors, including breast cancer, lung cancer, and head and neck tumors [8–10]. In addition, total body irradiation (TBI) has been widely used as a conditioning regimen for patients who require the treatment for bone marrow transplantation [11]. However, radiation-induced activation of the immune system has been increasingly recognized in recent years, an indication that RT could also elicit immune-mediated anti-tumor responses. In fact, the role of T cells in local tumor control induced by RT was demonstrated in a murine fibrosarcoma model over 30 years ago. The required radiation dose to control 50% of the tumors was much lower in immunocompetent mice compared to that of T cell-deficient mice (30 gray [Gy] vs. 64.5 Gy), and immunocompetent mice also had a lower incidence of metastases than immunosuppressed mice [12]. Similarly, in mouse melanoma tumor models, Lee et al. demonstrated that only immunocompetent hosts responded to 15–20 Gy radiation, while nude mice lacking T cells and B cells and wild-type mice depleted of CD8+ T cells did not respond to this high-dose radiation [13]. In patients, Holecek and Harwood reported that one Kaposi’s sarcoma patient who previously received a kidney transplant and was treated with azathioprine to suppress kidney rejection responded less to irradiation than those who did not receive an exogenously administered immunosuppressive agent [14]. Furthermore, other studies have found that this immune-mediated anti-tumor effect of RT could also trigger the regression of metastatic tumors that were distant from the irradiated field, which is the so-called abscopal effect. This effect, initially defined by Mole in 1953 [15], was detected in renal cell carcinoma, melanoma, lymphomas, hepatocellular carcinoma, and other tumor types [16–23]. For instance, Stamell et al. reported a metastatic melanoma patient who received palliative RT to the primary tumor also experienced regression of non-irradiated metastases [17]. An abscopal effect has also been reported in mouse tumor models in which Demaria et al. observed that the abscopal effect was tumor-specific and only occurred in wild-type mice that were treated with a combination of RT and Flt3-L, a growth factor that stimulates the production of dendritic cells (DCs). But no growth delay of secondary non-irradiated tumors has been observed in immunodeficient athymic mice or in wild-type mice treated with single dose of RT alone, further confirming that the abscopal effect was mediated by immune mechanisms [24].

However, although the abscopal effect of RT alone has been reported by a growing number of trials and cases, the overall occurrence rate was relatively low. This may be explained by the insufficiency of RT alone to overcome the immunoresistance of malignant tumors. Given that immunotherapy can reduce host’s immune tolerance toward tumors, it is possible that the combination of RT and immunotherapy can amplify the anti-tumor immune response, which is more likely to cause the occurrence of an abscopal effect [25–27]. In fact, this synergistic anti-tumor effect has been investigated in many clinical studies (Table 1). Nevertheless, the mechanism of the abscopal effect is not yet completely understood. Therefore, in this review, we describe the anti-tumor effect of RT and immune checkpoint blockade and discuss several publications on the abscopal effect of combination therapy, primarily to define the potential predictors of this effect so that the appropriate patients could receive more appropriate treatment. In addition, the second aim of this review is to evaluate the optimal timing for coupling RT with immune checkpoint blockade and to determine the most effective dose and fractionation of RT in the context of combination treatments.

**Table 1:** Some related clinical studies of RT combined with immunotherapy

Authors	Years	Tumors	Numbers of cases	Immunotherapy	RT	Sequence of RT and immunotherapy	Occurrence of abscopal effect
Roger et al. [157]	2018	Melanoma	25	Anti-PD-1 (pembrolizumab, 2 mg/kg/3 weeks or nivolumab, 3 mg/kg/2 weeks)	26 Gy/3–5 fractions	Concurrent and post-radiation	Observed
Formenti et al. [135]	2018	Metastatic breast cancer	23	Anti-TGFβ (fresolimumab,1 mg/kg/3 weeks or 10 mg/kg/3 weeks)	22.5 Gy/3 fractions	Concurrent	Observed
Rodríguez-Ruiz et al. [136]	2018	Advanced cancer	15	DC vaccination and TLR-3 agonist	Stereotactic ablative RT	Concurrent	Observed
Aboudaram et al. [130]	2017	Melanoma	17	Anti-PD-1 (pembrolizumab, 2 mg/kg/3 weeks or nivolumab, 3 mg/kg/3 weeks)	30 Gy/10 fractions	Concurrent	Observed
Theurich et al. [158]	2016	Melanoma	45	Anti-CTLA-4 (ipilimumab, 3 mg/kg/3 weeks)	SBRT	Concurrent and post-radiation	Observed
Koller et al. [119]	2016	Melanoma	70	Anti-CTLA-4 (ipilimumab, 3 mg/kg/3 weeks)	Conventional external beam radiation and stereotactic radiosurgery	Concurrent	Observed
Twyman-Saint et al. [145]	2015	Melanoma	22	Anti-CTLA-4 (ipilimumab)	Lung/bone 8 Gy × 2 or 8 Gy × 3 Liver/subcutaneous 6 Gy × 2 or 6 Gy × 3	RT before ipilimumab	Observed
Golden et al. [107]	2015	Metastatic solid tumors	41	GM-CSF (125 μg/m^2^/2 weeks)	35 Gy/10 fractions	Concurrent	Observed
Grimaldi et al. [118]	2014	Melanoma	21	Anti-CTLA-4 (ipilimumab, 3 mg/kg/3 weeks)	RT of brain metastasis or extracranial sites	RT after ipilimumab	Observed
Hwang et al. [159]	2018	Metastatic lung cancer	164	Anti-PD-1/PD-L1	Thoracic RT	RT before or after immunotherapy	Non-observed
Shaverdian et al. [129]	2017	NSCLC	97	Anti-PD-1 (pembrolizumab, 2 mg/kg/3 weeks, 10 mg/kg/3 weeks, or 10 mg/kg/3 weeks)	Extracranial radiotherapy and thoracic radiotherapy	Ipilimumab after RT	Non-observed
Kropp et al. [160]	2016	Melanoma	16	Anti-CTLA-4 (ipilimumab, 3 mg/kg/3 weeks)	SBRT	RT after ipilimumab	Non-observed
Levy et al. [133]	2016	Metastatic tumors	10	Anti-PD-L1 (durvalumab, 10 mg/kg/3 weeks)	28 Gy/5 fractions (median)	Concurrent	Non-observed
Kwon et al. [121]	2014	Castration-resistant prostate cancer	799	Anti-CTLA-4 (ipilimumab, 10 mg/kg/3 weeks)	8 Gy/target bone lesion	Ipilimumab after RT	Non-observed
Slovin et al. [120]	2013	Castration-resistant prostate cancer	50	Anti-CTLA-4 (ipilimumab, 10 mg/kg/3 weeks)	8 Gy/target bone lesion	Concurrent	Non-observed

*RT* radiotherapy, *NSCLC* non-small cell lung cancer, *GM-CSF* granulocyte-macrophage colony-stimulating factor, *SBRT* stereotactic body radiotherapy

## RT reprograms the tumor microenvironment

Under the selective pressure of the immune system, cancer cells have evolved a series of immune resistance mechanisms to escape the elimination of the anti-tumor immune responses, which is known as immunoediting [28, 29]. Some tumors lack the appropriate inflammatory cytokines and chemokines to attract immune cells, such as DCs, macrophages, and cytotoxic T cells, to the tumor site, and the expression of immunosuppressive ligands and death ligands inhibits the function and the activation of T cells. In addition, the downregulation of adhesion molecules, such as vascular cell adhesion molecule 1 (VCAM1) and intercellular adhesion molecule 1 (ICAM1), leads to an enhancement of a tumor vasculature barrier that inhibits T cell arrest and transmigration. Along with other immunosuppressive factors, such as the existence of inhibitory immune cells and the downregulation of the major histocompatibility complex (MHC), these complex interaction mechanisms contribute to cancer cell escape [30, 31]. However, although these immune escape mechanisms lead to the growth and invasion of tumors, the immune system can still recognize and clear tumor cells, and interventions such as RT that can promote the release of tumor neoantigens may potentially lead to effective immune responses and cancer control. Importantly, under certain conditions, RT can reprogram the anti-immunologic tumor microenvironment, making it more conducive for antigen-presenting cells (APCs) and T cells to recruit and function, thereby inducing tumor cells to be recognized and eradicated more easily by the immune system.

### Radiation-induced release of cytokines and chemokines

Localized radiation induces a burst release of cytokines and chemokines, giving rise to an inflammatory tumor microenvironment. These factors are secreted by irradiated tumor cells and other cells such as fibroblasts, myeloid cells, and macrophages. Various types of cytokines and chemokines play different roles in modulating the immune response, either pro- or anti-immunogenic, and maintain a net balance in the tumor milieu.

Radiation-induced interferons (IFNs), which represent the main effector molecules of the anti-tumor immune response, play a significant role in the therapeutic effect of RT. The induction of type I IFN by RT is essential for the activation and function of DCs and T cells, which, in turn, is responsible for the release of IFN-γ and tumor control [32, 33]. IFN-γ (type II IFN) acts on tumor cells to induce the upregulation of VCAM-1 and MHC-I expression, thereby enhancing the presentation of tumor antigens [34]. Indeed, type I IFN non-responsive mice showed an abolished anti-tumor effect of RT, and an exogenous increase in type I IFN could mimic the therapeutic effect of RT on tumor regression [32]. The production of type I IFN after irradiation is mediated by the stimulator of interferon genes (STING) and its upstream cyclic guanosine monophosphate-adenosine monophosphate synthase (cGAS) signaling pathways by sensing cancer cell-derived cytosolic DNA [35]. This process can be detected in both cancer cells and in infiltrating DCs [36]. However, high-dose radiation, specifically a single dose above a threshold ranging from 12 to 18 Gy, would induce upregulation of the three prime repair exonuclease 1 (Trex 1) in tumor cells. Trex 1 is a DNA nuclease which can degrade cytoplasmic DNA and in turn preclude the induction of type I IFN mediated by the activation of the cGAS-STING pathway, demonstrating the radiation dose dependency of the activation of type I IFN signaling [37, 38].

Transforming growth factor beta (TGFβ), acting as a major immunosuppressive factor, is also released and activated during RT [39]. This radiation-induced pleiotropic cytokine is important in regulating tissue homeostasis in the tumor microenvironment that inhibits the immune response by reducing the antigen-presenting ability of DCs and the activation of effector T cells [40]. In addition, TGFβ also causes radioresistance of tumor cells and reduces their radiosensitivity [41]. Taken together, the RT-mediated release of TGFβ promotes tumorigenesis and metastasis and leads to poor clinical outcomes for patients [42].

The release of other radiation-induced cytokines in the tumor microenvironment also influences the delicate balance between immune clearance and immune tolerance. For instance, the induction of interleukin-6 (IL-6), IL-10, and colony stimulating factor 1 (CSF-1) contributes to the proliferation and invasion of tumor cells and thereby displays a pro-tumorigenic role [43–46]. In contrast, the secretion of pro-inflammatory IL-1β enhances the anti-tumor immune response [47, 48]. Furthermore, the differential expression of RT-induced chemokines determines the type of leukocyte infiltration in the tumor microenvironment. For example, the production of CXC-motif chemokine ligand 12 (CXCL12) results in chemotaxis of pro-tumorigenic CD11b+ myeloid-derived cells [49], whereas the upregulation of CXCL9, CXCL10, and CXCL16 can attract anti-tumor effector T cells [50–52]. These conflicting mechanisms reflect the complexity of the tumor microenvironment.

### Radiation-induced infiltration of leukocytes

The radiation-induced release of inflammatory cytokines and chemokines increases tumor infiltration by various leukocytes including not only leukocytes that enhance anti-tumor immune responses, such as DCs, effector T cells, and natural killer (NK) cells [53–55], but also immunosuppressive cells such as regulatory T cells (Treg cells) and CD11b+ cells, including myeloid-derived suppressor cells (MDSCs) and tumor-associated macrophages (TAMs) [56–59].

RT can induce the maturation of DCs and facilitate their migration to draining lymph nodes. These migratory tumor-associated DCs are important in the presentation of tumor antigens, which endogenously trigger the priming of antigen-specific effector T cells and their subsequent infiltration into tumors [53, 54]. In addition, radiation-induced normalization of the vasculature allows for more efficient infiltration of effector T cells [60]. In fact, the presence of tumor-infiltrating T cells has been shown to correlate with better clinical outcomes in patients with a variety of cancers such as colorectal cancer, ovarian cancer, and breast cancer [61–63]. In addition, NK cell-mediated cytotoxicity also plays a significant role in eliminating tumor cells, which can be enhanced by RT since radiation increases the expression of tumor ligands for NK cell-activating receptors, such as NKG2D and NKp30 [64–66].

Treg cells are a special type of CD4+ T cells, and they play a key role in maintaining tumor immune tolerance. In the tumor microenvironment, accumulated Treg cells can secrete relative immunosuppressive cytokines such as TGFβ and IL-10, which impair the antigen-presenting function of DCs and the activation of effector T cells. In addition, Treg cells can also promote tumor angiogenesis and enhance MDSCs to exert their immunosuppressive function, eventually leading to tumor progression [67]. MDSCs are heterogeneous myeloid cells consisting of two major subsets: granulocytic MDSC (G-MDSC) and monocytic MDSC (M-MDSC) [68, 69]. Both populations contribute to tumor progression not only by their negative regulatory effects on the immune system but also by promoting tumor cell invasion and metastasis [70]. Many studies have reported the presence of increased numbers of Treg cells and MDSCs after RT in the tumor microenvironment, which is associated with poor prognosis in cancer patients [56, 57, 71].

Macrophages are another type of leukocyte that can infiltrate the tumor microenvironment. They can be described by two phenotypes, M1 and M2 macrophages, that have different functions [72]. The classical activation of M1 macrophages can induce the release of pro-inflammatory cytokines such as IL-12 and tumor necrosis factor (TNF) and play a role in killing tumor cells. In contrast, M2 macrophages act as anti-immunogenic cells that express anti-inflammatory cytokines such as IL-10 and TGFβ, which subsequently inhibit the function of effector T cells and favor tumor progression [73]. Indeed, most TAMs are tumor-promoting M2 macrophages [74]. Interestingly, in a pancreatic tumor model, Klug et al. have reported that low-dose irradiation could reprogram the differentiation of TAMs to an M1 phenotype and enhance anti-tumor immunity [75]. Further studies are required to elucidate the effect of RT on TAMs.

### Radiation-induced increased susceptibility of tumor cells

RT can also increase the susceptibility of tumor cells to immune-mediated tumor rejection. Upregulation of MHC-I molecules after RT has been observed in many studies. For example, Reits et al. observed that ionizing radiation, particularly at higher doses (10–26 Gy), could enhance the expression of MHC-I in a dose-dependent manner in both in vitro and in vivo studies, which increased the presentation of tumor antigens and rendered tumor cells more susceptible to T cell attack [76]. In addition, RT can induce the expression of Fas and ICAM-1 on tumor cells, rendering them more sensitive to T cell-mediated lysis, which can be blocked by the administration of anti-FasL [77]. Nevertheless, RT can also upregulate the expression of negative immune checkpoint ligands such as programmed death-ligand 1 (PD-L1) and impair the anti-tumor immune responses of effector T cells [78, 79]. Therefore, the influence of RT on the tumor microenvironment is very complex because of its dual effects on the host immune system. These opposing mechanisms for radiation are summarized in Table 2.

**Table 2:** The dual effects of RT on tumor microenvironment

Effect of RT	Pro-immunogenic	Anti-immunogenic
Cytokine secretion	IFN I	TGF-β
	IFN II	CSF-1
	IL-1β	IL-6
	IL-18	IL-10
Chemokine secretion	CXCL9	CXCL12
	CXCL10	
	CXCL16	
Leukocyte infiltration	DCs	MDSCs
	Effector T cells	Treg cells
	M1 macrophages	M2 macrophages
Signal molecule expression	MHC-I	PD-L1
	STING	Trex 1
	Fas	

*RT* radiotherapy, *IFN* interferon, *IL* interleukin, *TGF* transforming growth factor, *CSF* colony-stimulating factor, *CXCL* CXC-motif chemokine ligand, *DCs* dendritic cells, *MDSCs* myeloid-derived suppressor cells, *Treg* regulatory T lymphocytes, *MHC* major histocompatibility complex, *STING* stimulator of interferon genes, *Trex* three prime repair exonuclease, *PD-L1* programmed cell death-ligand 1

## Anti-tumor immune effects of RT: from local to abscopal

### RT generates in situ vaccination

RT can promote a special functional type of cell apoptosis named immunogenic cell death (ICD) [80–82] and can stimulate antigen-specific, adaptive immunity by some undetermined mechanisms [83]. ICD leads to subsequent anti-tumor immune responses including the release of tumor antigens by irradiated tumor cells, the cross-presentation of tumor-derived antigens to T cells by APCs, and the migration of effector T cells from the lymph nodes to distant tumor sites. These processes illustrate that irradiated tumors can act as an in situ vaccination [82, 84, 85].

Due to the stress response that is induced by irradiation, the dying tumor cells experience a series of subtle changes involving the pre-apoptotic translocation of endoplasmic reticulum (ER) proteins, such as calreticulin (CRT) [82, 86], from the ER to the cell surface, and the release of damage-associated molecular pattern molecules (DAMPs) [87], such as high-mobility group box 1 (HMGB1) [88] and adenosine triphosphate (ATP) [89, 90] from the cytoplasm of stressed tumor cells to the outside environment. CRT, acting as an “eat-me” signal, promotes the uptake of irradiated tumor cells by APCs such as DCs and phagocytic cells [86, 90–92]. The release of DAMPs, including HMGB1 and ATP, is another characteristic change that occurs during cell death after exposure to radiation [93, 94]. Acting as a “find-me” signal to recruit APCs [95], ATP can attract monocytes and DCs to tumors by a purinergic receptor P2X7-dependent pathway and promote the secretion of pro-inflammatory cytokines such as IL-1β and IL-18 [96, 97]. HMGB1 is a histone chromatin-binding protein [98], and when it binds to the surface pattern recognition receptors (PRRs), such as Toll-like receptor (TLR) 2 and TLR 4, it exerts its potential pro-inflammatory effect [94]. This interaction drives downstream inflammation responses and promotes the processing and presentation of tumor antigens by host APCs [94, 98]. Additionally, HMGB1 can also facilitate the maturation of DCs, thereby enabling them to present antigens efficiently to T cells, a process that is mediated by type I IFNs [57]. As mentioned before, the production of type I IFNs depends on the activation of the cGAS-STING pathway by sensing cancer cell-derived DNA and can be impaired by the DNA nuclease Trex 1 [37, 38]. All of these processes contribute to the effective presentation of tumor antigens by DCs and exert potent immunomodulatory effects.

DCs interact with tumor antigens and then migrate to the lymph nodes where they present these antigens to T cells, a process that is mediated by the MHC pathway via recognition by the T cell receptor (TCR). Furthermore, the basic leucine zipper ATF-like transcription factor 3 (BATF3)-dependent DC subset has been recently shown to be essential for the cross-priming of CD8+ T cells, which are key effectors in anti-tumor immunity. These DCs can take up tumor antigens effectively and introduce these antigens by way of the MHC class I cross-presenting pathway. Indeed, Batf3^−/−^ mice exhibit an impaired ability to cross-prime cytotoxic T lymphocytes against tumor antigens [99, 100].

**Fig. 1:**
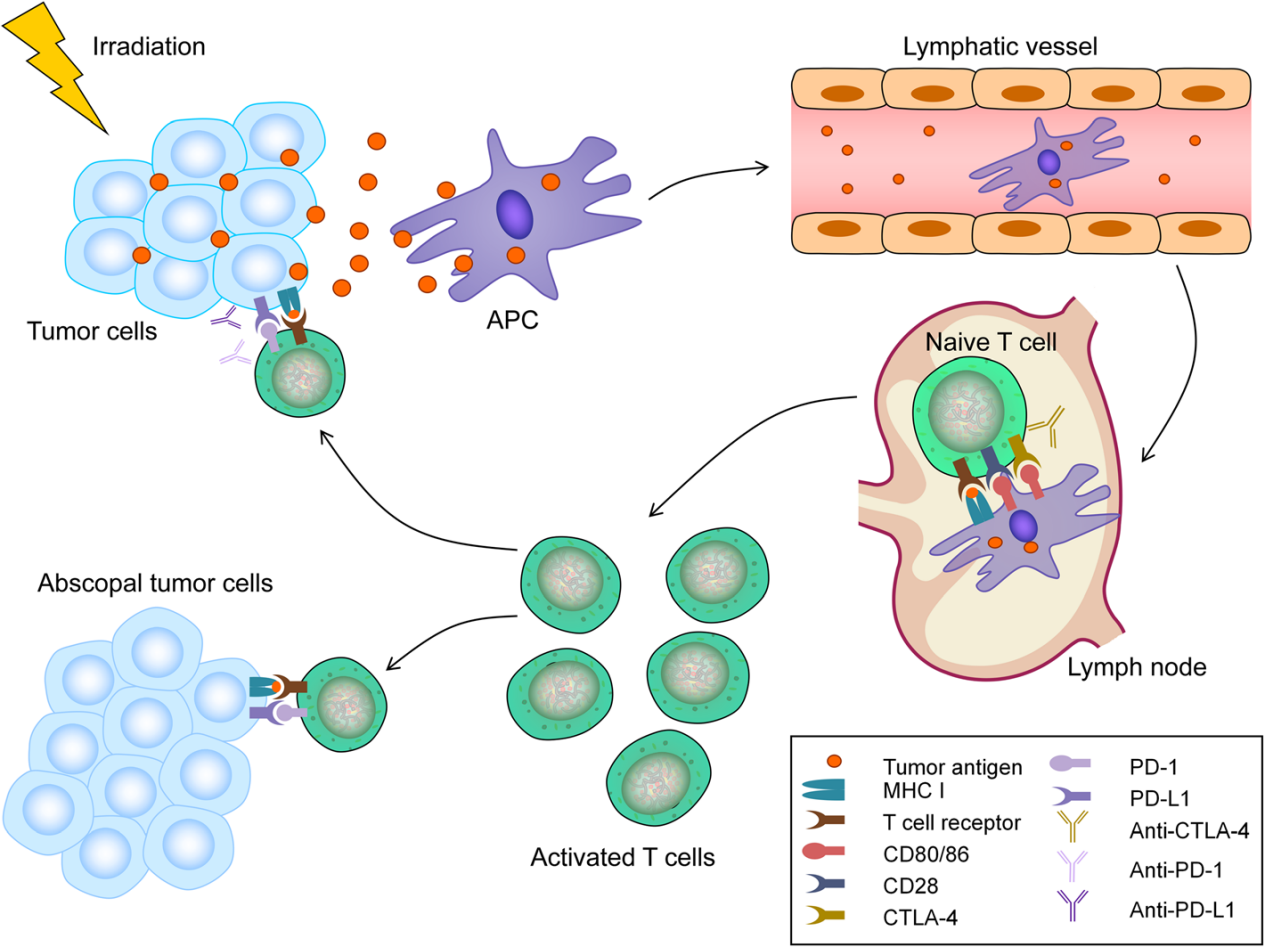
Mechanism of the abscopal effect. Radiotherapy (RT) can lead to immunogenic cell death and the release of tumor antigens by irradiated tumor cells. These neoantigens are taken up by antigen-presenting cells (APCs), such as dendritic cells (DCs) and phagocytic cells. The APCs interact with tumor antigens and then migrate to the lymph nodes where they present antigens to T cells, a process that is mediated by the MHC pathway and other co-stimulatory signals, such as CD80 and CD28. After activation by multiple signals, T cells, especially the CD8+ T cells, are activated and begin to propagate. As a result, activated effector T cells exit the lymph nodes and home to tumors, including primary tumors and non-irradiated tumor metastases, to exert their effect of killing tumor cells. However, cytotoxic T lymphocyte-associated antigen 4 (CTLA-4) competitively combines with CD80/86 and inhibits the activation of T cells. Following T cell activation, programmed cell death 1 (PD-1) receptors that are expressed on the T cell surface bind primarily to programmed death-ligand 1 (PD-L1) and inhibit immune responses. The administration of immune checkpoint blockades of CTLA-1, PD-1, and PD-L1 can enhance the anti-tumor immunity of RT

However, antigen-MHC complex interactions alone are insufficient to lead to the activation of T cells; other co-stimulatory signals such as CD80, CD40 L, and CD28 are also required [84]. After activation by multiple signals, T cells, especially the CD8+ T cells that play a major role in the anti-tumor immune response, are activated and begin to propagate. As a result, activated effector T cells exit the lymph nodes and home to tumors to exert their effect of killing tumor cells [101]. This mechanism can be used to explain the regression of distant metastatic tumor lesions combined with the locally irradiated tumors (Fig. 1). In fact, following the first report of the abscopal effect [15], the regression of distant tumor lesions after RT had been documented by many case reports of several malignant tumors such as melanoma, breast cancer, and lung cancer [18, 102, 103]. However, the overall incidence of the abscopal effect is low, and only 46 clinical cases of the abscopal effect due to RT alone have been reported from 1969 to 2014 [104]. This rare phenomenon can be explained by the insufficiency of RT alone to overcome the established immune tolerance mechanisms of tumor cells. Currently, many studies have shown that combining RT with immunotherapy can effectively overcome tumor immunosuppression and boost abscopal response rates compared with the use of RT alone [105–107].

### Immunotherapy enhances the systemic anti-tumor response of RT

#### CTLA-4 and CTLA-4 blockade

As previously mentioned, the activation of T cells requires an interaction between the TCR and a peptide-MHC complex with APCs, as well as a dynamic balance between the co-stimulatory and inhibitory signals that regulate the effectiveness of the immune response. Among them, the binding of CD28 on T cells with the B7 family ligands CD80 and CD86 that are located on APCs is the dominating co-stimulatory signal. Because another trans-membrane receptor, cytotoxic T lymphocyte-associated antigen 4 (CTLA-4), can also combine with CD80/86, it has been considered as one of the major negative immunomodulatory receptors that attenuate T cell activation [108–110] (Fig. 1). Therefore, the blockade of CTLA-4 is considered to be a promising immunotherapeutic method for enhancing the anti-tumor immune response, and a series of preclinical and clinical trials have demonstrated the anti-tumor effect of the CTLA-4 blockade in solid tumors, largely in patients with malignant melanoma. For example, two clinical trials have demonstrated that treatment of patients with advanced melanoma using anti-CTLA-4 (ipilimumab) could lead to durable responses and improve the overall survival of patients [111, 112]. Furthermore, patients with ovarian cancer, prostate cancer, and renal cell carcinoma could also benefit from anti-CTLA-4 immunotherapy [113–115].

However, the anti-tumor effect of CTLA-4 blockade alone is limited, and monotherapy may lead to serious autoimmune-related side effects such as dermatitis, colitis, hepatitis, and hypophysitis [116]. Given that blocking CTLA-4 could enhance the activation of T cells and increase the ratio of CD8+ T cells to Treg cells [117], which can strengthen the in situ vaccination effect of RT [110], the combined application of ipilimumab with RT has been increasingly valued by researchers and clinicians. In fact, this combination treatment strategy has achieved encouraging results in studies in both mice and humans and has been approved for the treatment of metastatic melanoma by the US Food and Drug Administration [111]. In a retrospective study, Grimaldi et al. documented a promising outcome for advanced melanoma patients treated with ipilimumab followed by RT. Among 21 patients, 11 patients (52%) experienced the abscopal effect, including 9 that had a partial response (PR) and 2 that had stable disease (SD). The median overall survival (OS) for patients with the abscopal effect was 22.4 months vs. 8.3 months for patients who did not experience this effect [118]. Consistently, in another retrospective analysis, Koller et al. demonstrated that advanced melanoma patients who received ipilimumab in combination with concurrent RT had a significantly increased median OS and complete response rates compared to those who did not [119]. Additionally, in a phase I/II study, Slovin et al. compared ipilimumab monotherapy with ipilimumab combined with RT (single fraction of 8 Gy) for patients with metastatic castration-resistant prostate cancer (mCRPC). The outcome was positive, in that among the 10 patients who received combination therapy, 1 had a PR and 6 had SD, and this combined approach of CTLA-4 blockade and RT could lead to durable disease control of mCRPC [120].

However, the outcomes were not always positive. In a clinical phase III trial, Kwon et al. also investigated the benefit of combination therapy with ipilimumab and RT in patients with mCRPC. Surprisingly, there were no differences in the median OS for the ipilimumab group compared to the placebo group, although reductions in prostate-specific antigen (PSA) concentration and improved progression-free survival (PFS) with ipilimumab treatment have been observed [121]. Therefore, additional studies are required to address this undetermined synergistic anti-tumor activity of combining RT with CTLA-4 blockade.

#### PD-1/PD-L1 and PD-1/PD-L1 blockade

Another co-inhibitory molecule, the inhibitory immune receptor programmed cell death 1 (PD-1), is expressed on the plasma membranes of T cells, DCs, and NK cells. PD-1 interferes with T cell-mediated signaling primarily through interactions with its two cognate ligands, PD-L1 and PD-L2, which are expressed by tumor cells. In fact, the expression of PD-L1 is upregulated in tumor cells, and PD-1 ligation by PD-L1 mainly promotes T cell apoptosis and leads to the elimination of activated T cells, thereby protecting tumor cells from T cell recognition and elimination [122–125]. Importantly, the upregulation of PD-L1 can be observed in experimental mouse tumor models after exposure to hypofractionated RT, which plays a key role in the RT resistance mechanism of tumor cells [79]. Consequently, we can hypothesize that the combination of the PD-1/PD-L1 blockade and RT may overcome tumor immunosuppression and improve the systemic effect of RT (Fig. 1). In fact, anti-PD-1/PD-L1 monoclonal antibodies (mAbs) have shown promising results in the treatment of non-small cell lung cancer (NSCLC), melanoma, and kidney cancer [126]. Additionally, two immune checkpoint inhibitors of PD-1, pembrolizumab and nivolumab, were approved by the US Food and Drug Administration for clinical application in patients with metastatic melanoma who experienced disease progression after prior treatment [127, 128].

In a secondary analysis of the KEYNOTE-001 phase trial, Shaverdian et al. assessed 97 advanced NSCLC patients who were treated with pembrolizumab. Patients who previously received RT achieved a significantly longer PFS (hazard ratio [HR] 0.56, *p* = 0.019; median PFS 4.4 vs. 2.1 months) and OS (HR 0.58, *p* = 0.026; median OS 10.7 vs. 5.3 months) than patients who did not previously receive RT [129]. Similarly, in a retrospective collection of consecutive patients with metastatic melanoma and who received PD-1 immune checkpoint inhibitors, Aboudaram et al. compared the survival data, overall response rates, and acute and delayed toxicities between patients receiving concurrent irradiation (IR) or no irradiation (NIR). Among 59 patients who received PD-1 blockade, 17 received palliative RT with a mean dose of 30 Gy that were delivered in 10 fractions. The objective response rate, including complete and partial response rates, was significantly higher in the IR group versus the NIR group (64.7 vs. 33.3%, *p* = 0.02) after a 10-month median follow-up and one complete responder experienced an abscopal effect. The 6-month disease-free survival (DFS) and OS rates were marginally increased in the IR group versus the NIR group (64.7% vs. 49.7%, *p* = 0.32; 76.4% vs. 58.8%, *p* = 0.42, respectively). Furthermore, no additional side effects were observed in the IR group, and the combination treatment was well tolerated [130]. In addition, abscopal effects have also been reported in patients with other malignant tumors, such as lung adenocarcinoma and Hodgkin’s lymphoma [131, 132]. However, in a single-center subset analysis from a phase I/II trial, Levy et al. reported that among 10 patients with metastatic tumors who received palliative local RT for 15 isolated lesions, the objective response (OR) rate was 60% after concurrent palliative RT and anti-PD-L1 durvalumab. Surprisingly, no outfield or abscopal effects were observed [133]. Therefore, although there are many encouraging reports concerning the combination of RT and anti-PD-1/PD-L1 mAbs, the rate of occurrence of abscopal effects is still undetermined. It is of significance to identify those patients who are most likely to respond, and additional or ongoing trials will hopefully elucidate their characteristics.

#### Other agents

Granulocyte-macrophage colony-stimulating factor (GM-CSF) is a potent stimulator of DC differentiation, proliferation, and maturation and facilitates the presentation of tumor antigens after cell death caused by RT [134]. In a prospective study conducted by Golden et al., the enrolled subjects were patients who had stable or advanced metastatic solid tumors after receiving single-agent chemotherapy or hormone therapy and had three distant measurable lesions. These patients were treated with RT (35 Gy in 10 fractions) to one metastatic site along with concurrent GM-CSF (125 μg/m^2^). In the space of 9 years, abscopal effects were observed in 11 of 41 accrued patients (specifically in 2 patients with thymic cancer, 4 with NSCLC, and 5 with breast cancer). In addition, the risk of death for patients without an abscopal effect was more than twice that of patients with it. This prospective clinical trial first demonstrated that an abscopal effect could provide patients with a better survival benefit and suggested a promising combination of RT with GM-CSF to establish an in-site anti-tumor vaccine [107].

Other immunotherapy modalities are still under investigation. Recently, Formenti et al. examined the role of anti-TGFβ therapeutics during RT to induce an abscopal effect in metastatic breast cancer patients. Fresolimumab, a TGFβ-blocking antibody, was administered in two doses, along with focal radiation of 22.5 Gy in three fractions. Although there was a general lack of abscopal effects, patients who received a higher fresolimumab dose had a significantly lower risk of death and a longer OS (median OS 16.00 vs. 7.57 months, *p* = 0.039) than those receiving a lower dose [135]. In addition, in another phase I clinical trial, Rodríguez-Ruiz et al. evaluated an intensive treatment modality in advanced cancer patients, which combined RT with two immune interventions, namely, intradermal DC vaccinations and intratumoral injections of Hiltonol, a TLR-3 agonist that can activate elements of both innate and adaptive immunity. The results demonstrated that this combined treatment was well tolerated, and one prostate cancer patient experienced an abscopal response [136]. Many other immunotherapeutic agents such as agonistic CD40 mAb and anti-galectin-1 may also boost abscopal effects by targeting different aspects of the immune-mediated response [137, 138]. In summary, combining these cancer immunotherapy modalities with standard-of-care chemoradiotherapy is a new frontier for future cancer treatment that may provide better efficacy. A brief summary of the representative ongoing clinical trials concerning the combination treatment of RT and immunotherapy is shown in Table 3.

**Table 3:** Representative ongoing clinical trials using CTLA-4/PD-1/PD-L1 inhibitors and RT for malignant tumors

ClinicalTrials.gov identifier	Phase	Conditions	Drug classification	Interventions	Sponsors
NCT01996202	Phase 1	Melanoma	CTLA-4 inhibitors	Ipilimumab with radiation therapy	Duke University
NCT02642809	Phase 1	EC	PD-1 inhibitors	Pembrolizumab with brachytherapy (16 Gy in 2 fractions)	Washington University School of Medicine
NCT02837263	Phase 1	Colorectal cancer	PD-1 inhibitors	Pembrolizumab with SBRT (40–60 Gy in 5 fractions)	University of Wisconsin, Madison
NCT02587455	Phase 1	Thoracic tumors	PD-1 inhibitors	Arm I: pembrolizumab with low-dose radiation therapy Arm II: pembrolizumab with high-dose radiation therapy	Royal Marsden NHS Foundation Trust
NCT03151447	Phase 1	TNBC	PD-L1 inhibitors	JS001 with SBRT	Fudan University
NCT02868632	Phase 1	Pancreatic cancer	PD-L1 and CTLA-4 inhibitors	Durvalumab or/and tremelimumab with SBRT (30 Gy in 5 fractions)	New York University School of Medicine
NCT03275597	Phase 1	NSCLC	PD-L1 and CTLA-4 inhibitors	Durvalumab and tremelimumab with SBRT (30–50 Gy in 5 fractions)	University of Wisconsin, Madison
NCT02239900	Phase 1/2	Liver cancer, lung cancer	CTLA-4 inhibitors	Ipilimumab with SBRT	M.D. Anderson Cancer Center
NCT03050554	Phase 1/2	NSCLC	PD-L1 inhibitors	Avelumab with SBRT (48 Gy in 4 fractions or 50 Gy in 5 fractions)	Andrew Sharabi
NCT02696993	Phase 1/2	Brain metastases (NSCLC)	PD-1 and CTLA-4 inhibitors	Arm I: nivolumab with stereotactic radiosurgery Arm II: nivolumab with whole brain radiation therapy Arm III: nivolumab and ipilimumab with stereotactic radiosurgeryArm IV: nivolumab and ipilimumab with whole brain radiation therapy	M.D. Anderson Cancer Center
NCT01970527	Phase 2	Melanoma	CTLA-4 inhibitors	Ipilimumab with SBRT	University of Washington
NCT02609503	Phase 2	Head and neck cancer	PD-1 inhibitors	Pembrolizumab with radiation therapy	UNC Lineberger Comprehensive Cancer Center
NCT02730130	Phase 2	Metastatic breast cancer	PD-1 inhibitors	Pembrolizumab with radiation therapy	Memorial Sloan Kettering Cancer Center
NCT02992912	Phase 2	Metastatic tumors	PD-L1 inhibitors	Atezolizumab with SBRT (45 Gy in 3 fractions)	Gustave Roussy, Cancer Campus, Grand Paris
NCT03122509	Phase 2	Metastatic colorectal cancer	PD-L1 and CTLA-4 inhibitors	Tremelimumab and durvalumab with radiation therapy	Memorial Sloan Kettering Cancer Center
NCT02888743	Phase 2	Colorectal cancer and NSCLC	PD-L1 and CTLA-4 inhibitors	Arm I: tremelimumab and durvalumab Arm II: tremelimumab and durvalumab with high-dose radiation therapy Arm III: tremelimumab and durvalumab with low-dose radiation therapy	National Cancer Institute (NCI)
NCT02701400	Phase 2	Recurrent SCLC	PD-L1 and CTLA-4 inhibitors	Arm I: tremelimumab and durvalumab Arm II: tremelimumab and durvalumab with SBRT	Emory University
NCT02617589	Phase 3	Brain Cancer	PD-1 inhibitors	Arm I: nivolumab with radiation therapy Arm II: temozolomide with radiation therapy	Bristol-Myers Squibb
NCT02768558	Phase 3	NSCLC	PD-1 inhibitors	Cisplatin and etoposide plus radiation followed by nivolumab	RTOG Foundation, Inc.

*SCLC* small cell lung cancer, *NSCLC* non-small cell lung cancer, *TNBC* triple-negative breast cancer, *EC* esophageal cancer, *SBRT* stereotactic body radiation therapy

## Future directions to improve abscopal effects of RT

### Optimal dose and fractionation of RT in abscopal effects

There are three dominant schemes of RT: conventional fractionation schemes (1.8~2.2 Gy/fraction, one fraction/day, 5 days/week for 3~7 weeks), hypofractionation including stereotactic radiosurgery (3~20 Gy/fraction, one fraction/day), and hyperfractionation (0.5~2.2 Gy/fraction, two fractions/day, 2~5 fractions/week for 2~4 weeks). The dose and fractionation of RT can influence its modulatory effects on the immune system, but it is worth noting that immunological effects of different regimens are unpredictable. Given that repetitive daily delivery of irradiation can kill migrating immune lymphocytes, Siva et al. believe that conventional fractionation schemes of RT are negative for radiation-induced anti-tumor immune responses. Their group also determined that single high-dose (12 Gy) RT did not deplete established immune effector cells such as CD8+ T cells and NK cells and that it might be much more efficient to kill tumor cells when combined with immunotherapy [139]. Indeed, compared with conventional modalities, RT with ablative high-dose per fractionation has been considered as a better treatment protocol to enhance the anti-tumor immune response [140]. Furthermore, in murine breast and colon cancer models, Dewan et al. showed that 5 × 6 Gy and 3 × 8 Gy protocols of RT were more effective in inducing immune-mediated abscopal effects than a single ablative dose of 20 Gy when combined with anti-CTLA-4 hamster mAbs 9H10 [141]. Similarly, in a murine melanoma model, Schaue et al. found that fractionated treatment with medium-size radiation doses of 7.5 Gy/fraction produced the best tumor control and anti-tumor immune responses [142]. Based on these experiences, many clinical trials aiming to evaluate the systematic anti-tumor effect of combinatorial immunotherapy and RT are designed with hypofractionated RT. It is encouraging that some of these studies have achieved satisfactory results and have observed the occurrence of abscopal effects. However, although larger doses per fraction may boost abscopal responses, other clinical studies did not achieve good outcomes, implying that abscopal effects are influenced by multiple factors (Table 1). Based on the dose and the fractionation of RT, an optimal threshold or range of doses is likely to exist. In a recent study, Vanpouille-Box et al. found that a radiation dose above a threshold of 10–12 Gy per fraction could attenuate the immunogenicity of cancer cells because of the induced upregulation of the DNA nuclease Trex 1, which can degrade cytoplasmic DNA and inhibit immune activation [37]. Thus, researchers should take these different data into a careful consideration in order to develop an optimal dose and fractionation scheme for RT in the context of radioimmunotherapy combinations to induce anti-tumor abscopal effects efficiently.

### Combination time window for RT and immunotherapy

The optimal schedule for the administration of RT relative to the immune checkpoint inhibitors is currently unclear. Should immune inhibitors of checkpoints be given concomitantly or sequentially with RT, and in which order? This time window may significantly influence the therapeutic anti-tumor response of this combination treatment.

Indeed, different combinatorial schedules have been evaluated in some preclinical studies. For instance, in mouse colon carcinoma models, in which a fractionated RT cycle of 2 Gy × 5 fractions was administered, Dovedi et al. evaluated three different schedules including the administration of anti-PD-L1 mAbs on day 1 of the RT cycle (schedule A), day 5 of the cycle (schedule B), or 7 days after the completion of RT (schedule C). Interestingly, both schedule A and schedule B achieved increased OS compared with RT alone, and there was no significant difference in the OS between these two subgroups. In contrast, sequential treatments with delayed administration of anti-PD-L1 mAbs at 7 days after RT completion (schedule C) were completely ineffective for improving the OS when compared with RT alone [143]. Similarly, in a murine breast model, Dewan et al. showed that the administration of anti-CTLA-4 mAbs at 2 days before or on the day of RT achieved a better therapeutic efficacy when compared with the delayed administration of mAbs at 2 days after RT [141]. Furthermore, some clinical case reports also imply the optimal time window of combining RT with immunotherapy. Golden et al. reported an abscopal effect in a treatment-refractory lung cancer patient treated with four three-weekly cycles of ipilimumab (3 mg/kg) and concurrent RT [144]. In addition, in a melanoma patient, Stamell et al. also observed an abscopal effect after combining ipilimumab with stereotactic RT concurrently [17]. Similarly, in the published clinical studies of radioimmunotherapy combinations, abscopal effects were mostly reported in patients who received RT while receiving concomitant immunotherapy (Table 1). Given the experience of preclinical and clinical trials in which abscopal effects were observed, although there is no consensus yet, the administration of immunotherapy initiated before or at the time of delivering RT may be preferred. However, in a phase I clinical trial of 22 advanced melanoma patients, Twyman-Saint et al. found that hypofractionated radiation followed by a treatment with the anti-CTLA4 antibody ipilimumab could also lead to partial responses in the non-irradiated lesions [145]. In addition, the potential toxicity of combination therapy, especially combinatorial radioimmunotherapy with concurrent regimens, limits their clinical application and should be investigated in further studies.

### Biomarkers for predicting the abscopal effect

Although a combination of immunotherapy and RT has achieved promising results in multiple solid tumors, not all of the patients experienced an abscopal effect. Therefore, it is necessary to identify efficient and effective biomarkers that can predict abscopal responses in patients who received combinatorial therapeutic regimens of immunotherapy and RT. In addition, validated biomarkers would be helpful in selecting suitable patients, identifying optimal therapeutic strategies, and predicting treatment responses.

As a tumor suppressor gene, p53 plays an important role in regulating the proliferation, apoptosis, and DNA repair of tumor cells, and its encoded protein P53 is a transcription factor that influences the onset of the cell cycle. As a guardian of the genome, p53 can inhibit the growth of tumors by obstructing the replication of damaged DNA, which acts as a major culprit inducing the abnormal proliferation of tumor cells [146]. However, the probability of a p53 mutation is greater than 50% among patients with malignant tumors, and a mutant p53 would lose its ability to inhibit the proliferation of tumor cells. In recent years, many studies have revealed that the status of p53 could regulate the abscopal anti-tumor effect of RT. In a mouse model system, Strigari et al. demonstrated growth inhibition of non-irradiated wild-type p53 tumors after irradiation of 20 Gy or 10 Gy. However, no significant tumor growth delay was observed in non-irradiated p53-null tumors regardless of the dose delivered [147]. Consistently, Camphausen et al. observed a similar result, in that the abscopal anti-tumor effect was observed neither in p53-null mice nor in mice in which p53 was inhibited by pifithrin-α, a drug that can block the p53 pathway [148]. Therefore, we can hypothesize that p53-dependent signals might be responsible for the systemic anti-tumor effect of RT, and an evaluation of the status of p53 in vivo might be used to predict the possibility of the occurrence of abscopal effects for cancer patients treated with RT regimens and thus provide better treatment administration.

In the Grimaldi et al. report on advanced melanoma, an abscopal effect was observed in 11 patients who were treated with ipilimumab followed by RT. Importantly, all patients who achieved an immune-related abscopal effect displayed a local response to RT. Thus, it is reasonable to speculate that a local response to RT could be of use to prognosticate abscopal effects. Furthermore, patients with an abscopal effect had a significantly higher median absolute lymphocyte count (ALC) before RT than those without an abscopal response, implying that lymphocyte counts preceding RT might be another patient parameter that can predict the occurrence of the abscopal effect. Nevertheless, given the limited number of patients in this retrospective study, further investigations are required to evaluate the predictive role of the local response to RT and the ALC on systemic abscopal effects [118].

Calreticulin expression may act as another potential marker to predict the response to combination treatments. As mentioned above, the radiation-induced translocation of calreticulin would promote the uptake of irradiated tumor cells by APCs and enhance the killing effect of T cells [86]. Furthermore, knockdown of calreticulin would impair the T cell recognition of tumor cells [149]. Therefore, the expression of calreticulin after RT implies susceptibility of tumor cells to T cell killing and can be used as a biomarker for the response to immunotherapy and RT. In addition, a recent preclinical study indicated that Trex 1 can be used as a potential biomarker to guide the administration of an optimal dose and fractionation of RT, which would be helpful in providing a better combination treatment strategy that might overcome the immunosuppression of tumor cells and facilitate the occurrence of abscopal effects [37, 38].

In addition, other biomarkers for immunotherapy have also been widely investigated. For instance, the tumor mutation burden (TMB) is closely related to the anti-cancer effect of immune checkpoint inhibitors, and patients with a high mutation burden experienced a long-term clinical benefit [150–152]. The PD-L1 expression can serve as a potential biomarker for the prediction of response to immunotherapies that target PD-1/PD-L1 [153–156]. However, a predictive role for them in the systemic abscopal effects of combinatorial immunotherapy and RT has yet to be defined. Furthermore, no specific sensitive biomarkers have been determined that can exclusively predict the abscopal responses in patients who experienced combined treatment regimens, and this is still an active area that needs to be further investigated.

## Conclusion

The abscopal effects of RT have been extensively reported in preclinical and clinical studies, and irradiated tumor cell death can stimulate anti-tumor adaptive immunity by promoting the release of tumor antigens and the cross-presentation of tumor-derived antigens to T cells. However, it is difficult for RT alone to overcome the immunoresistance of malignant tumors. With the development of cancer immunotherapy, especially immune checkpoint inhibitors, the abscopal effect of RT has become more meaningful, since the in situ vaccination that is generated by RT can be substantially potentiated by immunotherapy. Exploiting the synergistic anti-tumor effect of these two treatments is encouraging because of its effective potential to improve the OS and PFS of patients with malignant tumors. However, many challenges remain for this combination treatment, including the determination of optimal dose/fractionation schemes for RT, the administration of optimal time points for these two treatment modalities, and the identification of relative biomarkers for the prediction of treatment efficacy. These challenges need to be addressed in future preclinical and clinical trials. In addition, translating these preclinical data into relevant and clinically efficient treatments and developing evidence-based consensus guidelines for RT and immunotherapy will also be required.

## Abbreviations

ALC: Absolute lymphocyte count
APCs: Antigen-presenting cells
ATP: Adenosine triphosphate
BATF3: Basic leucine zipper ATF-like transcription factor 3
cGAS: Cyclic guanosine monophosphate-adenosine monophosphate synthase
CRT: Calreticulin
CSF-1: Colony stimulating factor 1
CTLA-4: Cytotoxic T lymphocyte-associated antigen 4
CXCL12: CXC-motif chemokine ligand 12
DAMPs: Damage-associated molecular pattern molecules
DCs: Dendritic cells
DFS: Disease-free survival
DNA: Deoxyribonucleic acid
ER: Endoplasmic reticulum
GM-CSF: Granulocyte-macrophage colony-stimulating factor
G-MDSC: Granulocytic MDSC
Gy: Gray
HMGB1: High-mobility group box 1
ICAM1: Intercellular adhesion molecule 1
ICD: Immunogenic cell death
IFNs: Interferons
IL-6: Interleukin-6
IR: Irradiation
mAbs: Monoclonal antibodies
mCRPC: Metastatic castration-resistant prostate cancer
MDSCs: Myeloid-derived suppressor cells
MHC: Major histocompatibility complex
M-MDSC: Monocytic MDSC
NIR: No irradiation
NK cells: Natural killer cells
NSCLC: Non-small cell lung cancer
OR: Objective response
OS: Overall survival
PD-1: Programmed cell death 1
PD-L1: Programmed death-ligand 1
PD-L2: Programmed death-ligand 2
PFS: Progression-free survival
PR: Partial response
PRRs: Pattern recognition receptors
PSA: Prostate-specific antigen
RT: Radiotherapy
SD: Stable disease
STING: Stimulator of interferon genes
TAMs: Tumor-associated macrophages
TBI: Total body irradiation
TCR: T cell receptor
TGFβ: Transforming growth factor beta
TLR: Toll-like receptor
TMB: Tumor mutation burden
TNF: Tumor necrosis factor
Treg cells: Regulatory T cells
Trex 1: Three prime repair exonuclease 1
VCAM1: Vascular cell adhesion molecule 1
